# A pre-clinical validation plan to evaluate analytical sensitivities of molecular diagnostics such as BD MAX MDR-TB, Xpert MTB/Rif Ultra and FluoroType MTB

**DOI:** 10.1371/journal.pone.0227215

**Published:** 2020-01-07

**Authors:** Markus Beutler, Sara Plesnik, Marina Mihalic, Laura Olbrich, Norbert Heinrich, Samuel Schumacher, Michael Lindner, Ina Koch, Wolfgang Grasse, Christoph Metzger-Boddien, Sabine Hofmann-Thiel, Harald Hoffmann

**Affiliations:** 1 Institute of Microbiology and Laboratory Medicine, Department IML Red GmbH, WHO-Supranational Reference Laboratory of Tuberculosis, Munich-Gauting, Germany; 2 Division of Infectious Diseases and Tropical Medicine, LMU University Hospital, Munich, Germany; 3 German Centre of Research on Infectious Diseases (DZIF), Partner Site Munich, Germany; 4 Foundation for Innovative New Diagnostics, Geneva, Switzerland; 5 Asklepios Fachkliniken München-Gauting, Munich, Germany; 6 gerbion GmbH & Co. KG, Kornwestheim, Germany; 7 SYNLAB Gauting, SYNLAB Human Genetics Munich, Munich-Gauting, Germany; Wadsworth Center, UNITED STATES

## Abstract

Rapid diagnosis of tuberculosis (TB) and antibiotic resistances are imperative to initiate effective treatment and to stop transmission of the disease. A new generation of more sensitive, automated molecular TB diagnostic tests has been recently launched giving microbiologists more choice between several assays with the potential to detect resistance markers for rifampicin and isoniazid. In this study, we determined analytical sensitivities as 95% limits of detection (LoD_95_) for Xpert MTB/Rif Ultra (XP-Ultra) and BD-MAX MDR-TB (BD-MAX) as two representatives of the new test generation, in comparison to the conventional FluoroType MTB (FT-MTB). Test matrices used were physiological saline solution, human and a mucin-based artificial sputum (MUCAS) each spiked with *Mycobacterium tuberculosis* in declining culture- and qPCR-controlled concentrations. With BD-MAX, XP-Ultra, and FT-MTB, we measured LoD_95_^TB^ values of 2.1 cfu/ml (CI_95%_: 0.9–23.3), 3.1 cfu/ml (CI_95%_: 1.2–88.9), and 52.1 cfu/ml (CI_95%_: 16.7–664.4) in human sputum; of 6.3 cfu/ml (CI_95%_: 2.9–31.8), 1.5 cfu/ml (CI_95%_: 0.7–5.0), and 30.4 cfu/ml (CI_95%_: 17.4–60.7) in MUCAS; and of 2.3 cfu/ml (CI_95%_: 1.1–12.0), 11.5 cfu/ml (CI_95%_: 5.6–47.3), and 129.1 cfu/ml (CI_95%_: 82.8–273.8) in saline solution, respectively. LoD_95_ of resistance markers were 9 to 48 times higher compared to LoD_95_^TB^. BD-MAX and XP-Ultra have an equal and significantly increased analytical sensitivity compared to conventional tests. MUCAS resembled human sputum, while both yielded significantly different results than normal saline. MUCAS proved to be suitable for quality control of PCR assays for TB diagnostics.

## Introduction

With 10 million new and 1.6 million mortal cases in 2017, tuberculosis (TB) still remains the world’s leading cause of death among single pathogen infections [1]. Anti-TB drug resistance (DR), especially multi-drug-resistance (MDR), pose major challenges to end TB [2–4]. Multi-drug-resistance is defined as resistance towards the two most powerful first line drugs rifampicin (Rif) and isoniazid (Inh). In previous decades, DR-TB mainly resulted from insufficient treatment; however, MDR-TB transmission is long promoting its own pandemic with an estimated 558,000 cases annually [5]. Rapid diagnosis is key to early initiation of effective treatment and to interrupt further spread of resistant TB, but in reality less than a third of MDR-TB cases are detected and therefore not treated [6,7].

Nucleic acid amplification tests (NAAT) are the fastest tools to accurately diagnose TB on the day of sample collection. While early generations of CE marked commercial NAATs like ProbeTec ET DTB (DTB) (Becton-Dickinson, USA) and COBAS TaqMan MTB (Roche, Switzerland) had relatively low sensitivities, more recent generations like FluoroType MTB (FT-MTB, Hain Lifescience, Germany) and Xpert MTB/RIF (XP-MTB; Cepheid, USA) have improved in this respect, but still markedly lag behind culture [8–10]. The cartridge-based XP-MTB additionally identifies Rif-resistance as a marker of MDR-TB [11] and has significantly ameliorated notification of TB and MDR-TB worldwide [12,13]. Now, with the launch of Abbott RealTime MTB and MTB INH/RIF (Abbott, USA), BD MAX^™^ MDR-TB (BD-MAX, Beckton Dickinson, USA), and Xpert MTB/Rif Ultra (XP-Ultra, Cepheid, USA) (Rif only), a new generation of closed and virtually fully automated qPCR assays detecting DNA of TB bacteria as well as Rif and/or Inh resistance markers are available which, according to the manufacturers’, have sensitivities nearing that of culture [14–17].

The analytical sensitivity expressed as 95% percentile of the *limit of detection* (LoD_95_^TB^) is a key parameter for the evaluation of the performance of NAATs [14]. Several factors impact the LoD_95_^TB^, most importantly the matrix in which the bacteria are dispersed. Deionized water, normal saline or buffer are frequently experimentally used, but do not reflect the reality of diagnostics with clinical samples. Human sputum is the most frequently collected clinical specimen in TB diagnostics, which strongly differs in its complexity, composition, and consistency from water or saline and even from sample to sample. Therefore, LoD_95_^TB^ values provided by authors from different studies are neither comparable among one another nor do they necessarily reflect the situation of diagnostics. In order to standardize a sputum matrix, either tremendous volumes of human sputum need to be pooled and extensively validated for the absence of *Mycobacterium tuberculosis* complex (MTBC)-DNA, or artificial sputum (AS) can be used which is produced under controlled and standardized conditions. Three major types of AS have been developed based either on polyacrylamide, on methylcellulose, or on watery dispersions [18–20]. Unfortunately, none of these resembles natural human sputum in consistency, chemical properties or viscosity; therefore, they do not simulate a clinical diagnostic sample. In order overcome this challenge, we have recently developed a novel and infinitely reproducible mucin-based artificial sputum (MUCAS), which very closely resembles human sputum in all these aspects.

In this study, we aimed to measure and head-to-head compare the analytical sensitivity (LoD_95_^TB^) of XP-Ultra, BD-MAX and FT-MTB using physiological saline, human sputum and our novel MUCAS as test matrices using a standardized validation plan.

## Methods

### Preparation of test matrices

Pooled human sputum, normal saline and mucin based artificial sputum (MUCAS) were used as test matrices for the determination of LoD_95_^TB^. MUCAS consisted of a mucin-based matrix enriched with bacteria of the normal respiratory flora (*Neisseria lactamica*, *Streptococcus rubneri* and *Moraxella lincolnii*) and human mononuclear cells (hMNC). All test matrices were spiked with *M*. *tuberculosis* (MTB) bacteria.

### Mycobacterium tuberculosis

MTB reference strain H37Rv (ATCC 35829) and a pre-characterized clinical MDR-MTB isolate T837 were cultured on Loewenstein-Jensen (LJ) medium (Enclit, Germany) for 2–4 weeks at 36±1°C. Bacterial suspensions were prepared in 0.85% saline solution containing 2.5 g of 1 mm glass beads (Neolab, Germany) and vortexed for 1 min. After two sedimentation steps of 10 min each, the final supernatant was adjusted to a turbidity of McF 4.0 measured in a BD phoenix nephelometer by adding 0.85% saline solution. To remove bulks of bacteria, this suspension was filtered through a 5 μm PVDF filter (Merck-Millipore, USA). The final turbidity was adjusted to McF 0.5 and serial dilutions produced with 0.85% saline.

### Mucin based artificial sputum

MUCAS was produced following the procedures specified in the MUCAS patent (patent application pending in the name of IML red GmbH; application number: EP19165015.9). The main ingredients of MUCAS were: mucin from porcine stomach type II (Sigma-Aldrich, USA), Middlebrook 7H9 powder (BD, USA), salmon DNA (Sigma-Aldrich, USA) and glycerine (VWR Chemicals, USA). Each MUCAS LOT was controlled for the absence of MTB DNA by performing XP-Ultra tests from 1 ml MUCAS from three different aliquots following the instructions of the manufacturer.

### Human sputum

Around 500 fully anonymized non TB, left-over sputum samples from regular patient care were mixed and stored at -20°C until usage. It was impossible to identify individual patients. No clinical or personal data were collected, stored or used for any purposes. Patients consented to use their samples for bacteriological research on TB. The study was reviewed and approved by the ethics committee of the Ludwig-Maximilians-University Munich (Germany). 7 x 200 ml sputum samples were pooled and controlled for the absence of MTB DNA by performing one XP-Ultra test per pool. Four MTB-negative pools were united to one master pool, homogenized by vigorous shaking and aliquoted in 50 ml aliquots. MTBC negativity was verified for five representative aliquots by inoculation of LJ slants after decontamination following the NALC-NaOH standard protocol [21], and incubating the cultures for 8 weeks at 36±1°C. Aliquots were stored at -20°C until usage.

### LoD_95_^TB^ and LoD_95_^HR^ (H: isoniazid-, R: rifampicin-resistance)

**Fig 1:**
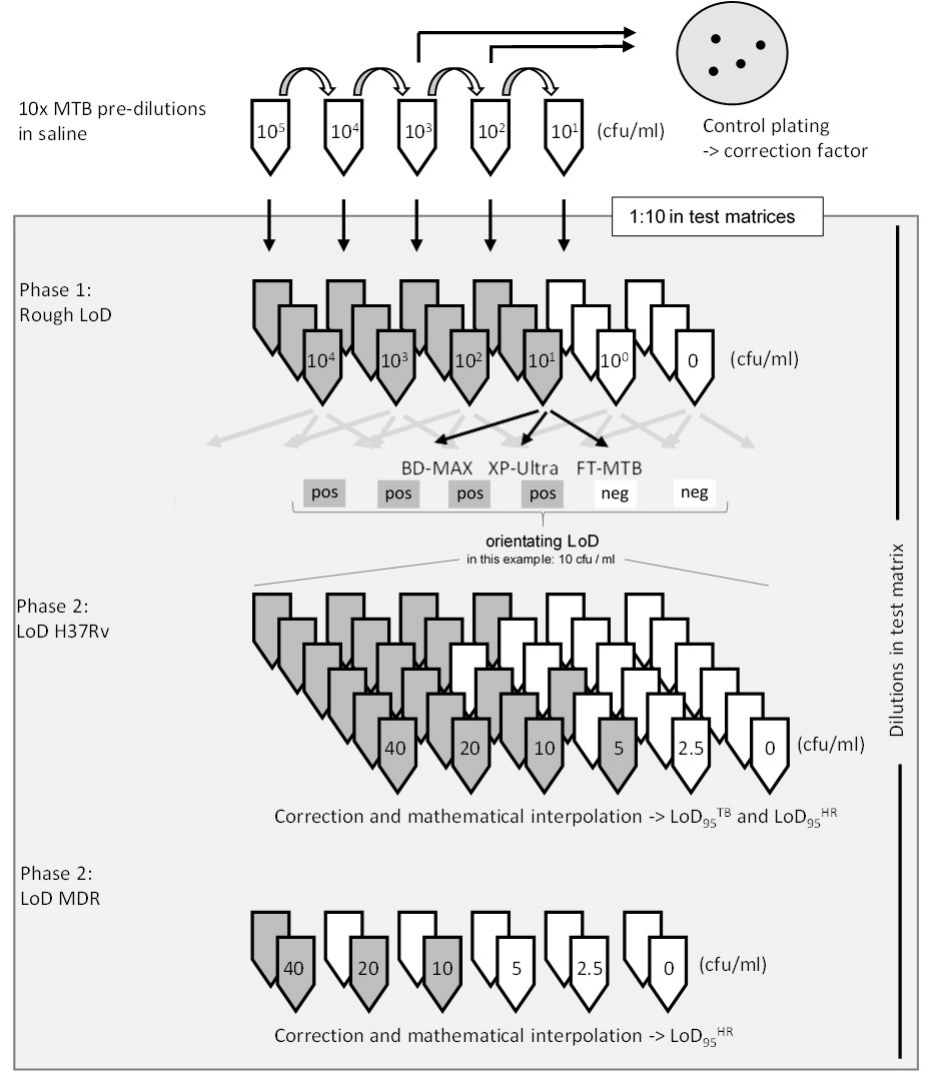
Schematic overview of LoD_95_ experiments. 10x pre-dilutions of H37Rv and an MDR MTBC isolate were prepared and spiked in human sputum, MUCAS or saline solution. In phase 1 three 10-fold dilution series (10^4^ to 0 cfu/ml) were produced in all test matrices. The orienting LoD was determined for each matrix/test combination (in the example, 10^0^ negative & 10^1^ positive = > LoD 10 cfu/ml). In phase 2 the LoD_95_^TB / HR^ were determined using at least six 2-fold dilutions per matrix/test around the respective orienting LoDs. Calculated LoD values were corrected according to colony counts of plates and interpolated using GraphPad Prism 8.0.2 to yield the 95% LoD and the 95%-CIs (GraphPad Software, USA).

LoD_95_ values were determined in two phases (Fig 1). In phase 1 the approximate LoD was determined for each target assay. 10x pre-dilutions of H37Rv bacteria were produced in 0.85% saline and spiked into the test matrices to reach final 10-fold dilution series ranging from 1x10^4^ to 1x10^0^ cfu/ml. In phase 2 the precise LoD_95_ values were measured by the use of 1:2 titer dilutions of suspensions of H37Rv bacteria spiked in the test matrices to reach six bacterial concentrations around the approximate LoD of each assay determined in phase 1. In the same way, dilutions of MDR-TB strain T837 were prepared around the target ranges of the assays to determine the LoD_95_^HR^ of the assays to detect Inh (H) and/or Rif (R) resistance markers. Control plating on 7H11 agar plates (incubation at 36±1°C for 3 weeks) were performed for each dilution series and the LoD_95_ values corrected according to the counted numbers of cfu. The LoD_95_, i.e. the bacterial concentration in the respective matrix that yielded positive results in 95% of test runs, was calculated using GraphPad Prism 8.0.2 (GraphPad Software, USA).

### Determination of cfu’s

Dilutions started with McF 0.5 corresponding to approximately 2.5 x 10^6^ cfu/ml of viable bacteria when processed according to our protocol, which is comparable to a previous study [22]. The exact number of cfu’s of each dilution series was determined by plating 100 μl of the suspensions with expected concentrations of 100 and 1000 cfu/ml on 7H11 agar plates and counting colonies after 3 weeks of incubation at 36±1°C. 900 μl aliquots of each suspension plated for colony counts were heat inactivated (30 min at 80°C) within 2 hours after production and stored at -20°C for maximum six months until quantitative IS*6110* PCR was performed as described below.

### BD-MAX, XP-Ultra, FT-MTB

All commercial PCR assays were CE marked and performed following to the manufacturer’s instructions:

#### BD-MAX and XP-Ultra

2 ml of either sample treatment reagent (BD-MAX) or sample reagent (XP-Ultra) were added to 1 ml spiked test matrix and inverted 10–20 times. After 5 min (BD-MAX) or 10 min (XP-Ultra) incubation, the samples were again inverted 10–20 times and further incubated for 25 min (BD-MAX) or 5 min (XP-Ultra) at room temperature. Samples were either transferred to BD-MAX tubes and sealed with a septum-cap or to XP-Ultra cartridges. The pre-treated samples together with the respective cartridges were loaded on the BD-MAX and GeneXpert machines and the programs were started following the algorithms of the respective platforms.

#### FT-MTB

1.0 ml of spiked test matrix was decontaminated with NALC-NaOH following the WHO standard protocol [21]. DNA extraction from 500 μl of decontaminated sample was performed using the FluoroLyse (Hain Lifescience, Germany). FT-MTB PCR mixes were prepared from AM-A and AM-B solutions and 6 μl of DNA was added. Positive (6 μl of C+ FT MTB) and negative controls (6 μl of FluoroLyse master-mix without spiked sample) were included in each run. PCRs were performed in FluoroCycler 12 instruments (Hain Lifescience, Germany) using the FluoroType Analysis software.

### Quantitative IS*6110* PCR and LoD_95_^IS6110^

The CE marked diarella MTB/NTM/MAC Kit (gerbion, Germany) and a synthetic IS*6110* DNA standard (gerbion, Germany) were used for qPCR to absolutely quantify numbers of IS*6110* insertion elements in test samples. A standard curve with tenfold dilutions ranging from 10^6^ to 10^0^ IS*6110* insertion elements/ml was produced and run in duplicates to determine PCR efficiency (result = 109%) and the dynamic range (down to 100 copies) of the assay. PCR reactions were executed on a Roche LightCycler 480 following the manufacturers’ instructions. Intensities of FAM (MTBC specific) and HEX (internal control) fluorescence were measured and recorded after each 60°C step. Colour compensation was performed using the *alpha*Cube LC480 Colour Compensation kit (Mikrogen Diagnostik, Germany). Numbers of IS*6110* insertion elements accessible for PCR were quantified by reproducing the initial standard curve produced with a single synthetic IS*6110* standard using the LightCycler software (Roche, Germany). For the determination of the LoD_95_^IS*6110*^, IS*6110* copies/ml were quantified for each H37Rv and T837 suspension with an expected bacterial concentration of 10^3^ cfu/ml. Individual cfu’s at the LoD in cfu/ml of each standard curve were determined and multiplied with the average IS*6110* copies/cfu. Furthermore, colony numbers counted on control plates were correlated to qPCR results. The resulting linear regression curve is presented in S1 Fig.

### Statistics and data analysis

Data were graphed and statistically analysed using GraphPad Prism 8.0.2. LoD_95_ values were interpolated and plotted with 95% confidence intervals. Differences between multiple groups were compared using one-way ANOVA with Tukey’s multiple comparison test. A p-value ≤ 0.05 was considered statistically significant. Numbers IS*6110* insertion per cfu were expressed as the geometric mean with 95% confidence interval.

## Results

### BD-MAX and XP-Ultra yield comparable analytical sensitivities

To comparatively investigate the analytical sensitivities of BD-MAX, XP-Ultra and FT-MTB, serial dilutions of H37Rv suspensions with controlled bacterial concentrations (in cfu/ml and IS*6110* copies/ml) were spiked in three test matrices: human sputum, MUCAS and 0.85% saline. The LoD_95_ of each assay was determined independently for each test matrix.

**Fig 2:**
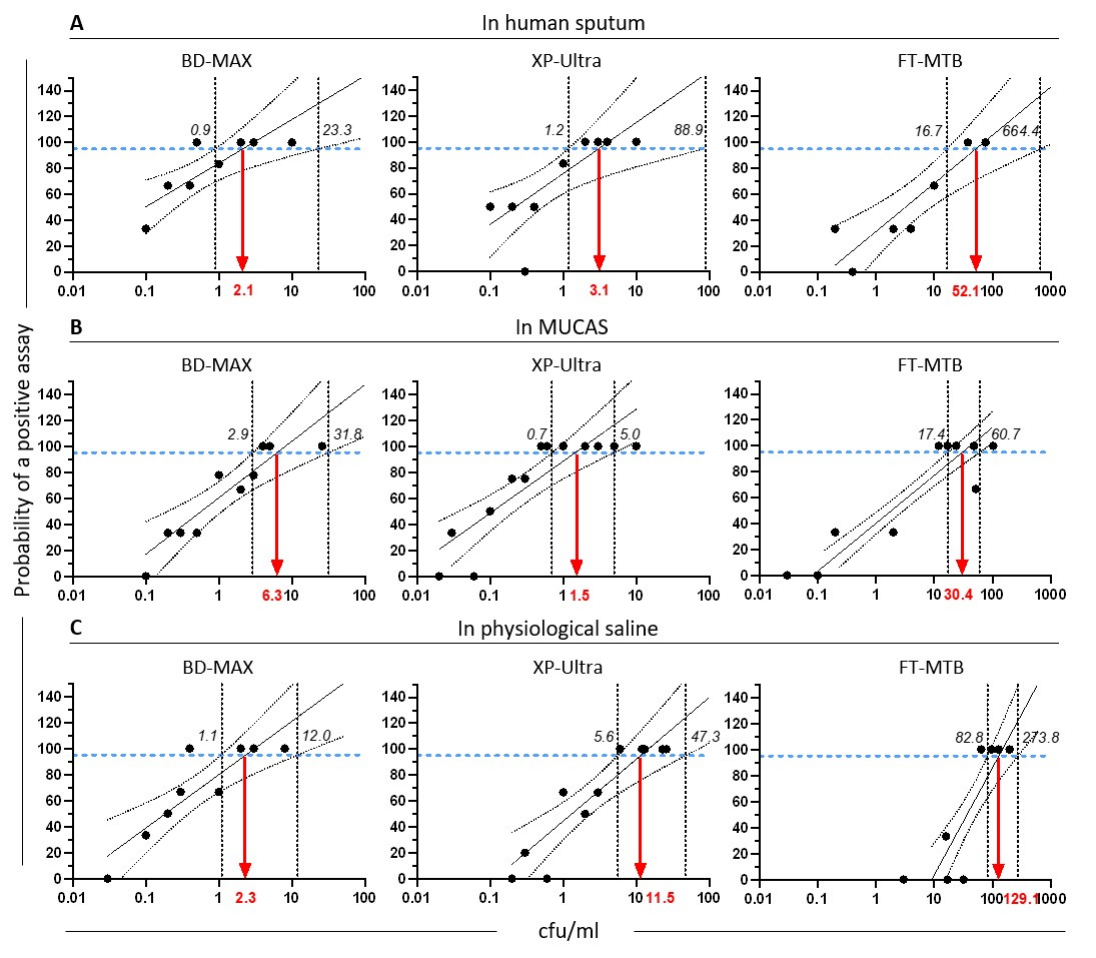
LoD_95_^TB^ of BD-MAX MDR-TB (BD-MAX), Xpert MTB/Rif Ultra (XP-Ultra) and FluoroType MTB (FT-MTB) in human sputum (A), MUCAS (B) and physiological saline solution (C). Red arrow: calculated LoD_95_^TB^, solid black line: predicted positive assay, blue dashed line: 95% probability of a positive assay, black dashed lines: upper and lower CI_95_ intervals.

The analytical sensitivities of BD-MAX and XP-Ultra were comparable in all three matrices and five to 56 times higher than those of FT-MTB (Fig 2). Values were interpolated from semi-log transformed curves. In both human sputum and MUCAS, BD-MAX (2.1 cfu/ml, CI_95%_: 0.9–23.3; and 6.3 cfu/ml, CI_95%_: 2.9–31.8; respectively) and XP-Ultra (3.1 cfu/ml, CI_95%_: 1.2–88.9; and 1.5 cfu/ml, CI_95%_: 0.7–5.0; respectively) yielded LoD_95_^TB^ values within similar though markedly lower ranges than FT-MTB (52.1 cfu/ml, CI_95%_: 16.7–664.4; and 30.4 cfu/ml, CI_95%_: 17.4–60.7; respectively). This difference became even more distinct in normal saline with which FT-MTB (129.1 cfu/ml, CI_95%_: 82.8–273.8) yielded a 11 to 56 times higher LoD_95_^TB^ than BD-MAX or XP-Ultra (2.3 cfu/ml, CI_95%_: 1.1–12.0; and 11.5 cfu/ml, CI_95%_: 5.6–47.3; respectively).

#### LoD_95_^IS*6110*^

While LoD_95_^TB^ was referenced to the number of bacteria determined as cfu/ml in quantitative cultures, LoD_95_^IS*6110*^ was referenced to copies of IS*6110* DNA in the solution. IS*6110* copies of H37Rv and MDR-TB strain T837 suspensions were determined using the CE marked commercial assay diarellaMTB/NTM/MAC Kit and the associated synthetic IS*6110* DNA standard (gerbion, Germany) which yielded an average of 11,777 IS*6110* copies/cfu of H37Rv (IC_95_: 5,743 to 24,150) and 2,900 IS*6110* copies/cfu of T837 (IC_95_: 339 to 24,815), suggesting that under *in vitro* conditions of spiked test matrices a large quantity of MTBC DNA occurs either as free molecules or bound to inanimate bacterial bodies.

**Fig 3:**
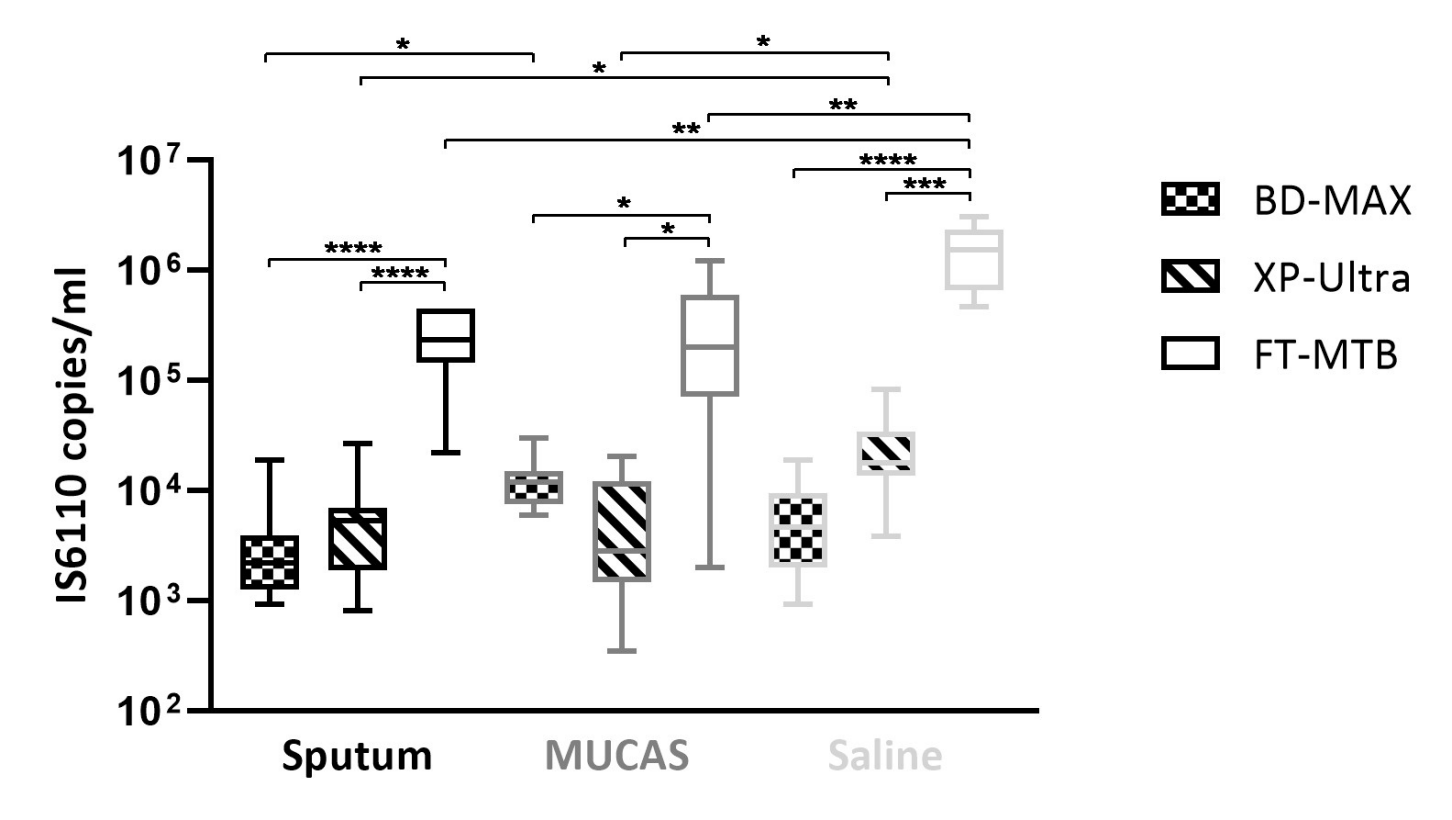
Whiskers Min to Max of IS*6110* copies at the limit of detection of BD-MAX, XP Ultra and FluoroType in different test matrices. Assays were performed in human sputum (Sputum), artificial sputum (MUCAS) and 0.85% saline solution (Saline). The limit of detection (LoD) was determined using two-fold dilutions of H37Rv spiked in test matrix. Number of test series of BD-MAX, XP-Ultra, FT-MTB in human sputum, MUCAS and saline were 8, 10, 6, and 8, 9, 9 and 9, 6, 9, respectively. **** p≤0.0001, *** p≤0.001, ** p≤0.01, * p≤0.05 (one-way ANOVA with Tukey’s multiple comparison test).

The LoD_95_^IS*6110*^ was defined as the limit of detection at which the concentration of MTB DNA in the respective matrix corresponded to the reported numbers of IS*6110* copies and the assay under investigation yielded 95% positive results. The distribution of LoD_95_^IS*6110*^ was determined in a total of 74 test series of two-fold dilutions of H37Rv bacteria in the respective test matrices, which are displayed in Fig 3 as Whisker min/max boxes around the respective medians. While LoD_95_^IS*6110*^ distributions of BD-MAX and XP-Ultra largely overlapped, LoD_95_^IS*6110*^ values of FT-MTB were approximately two logs higher in all three test media (p≤0.05 to p≤0.0001; one-way ANOVA). Investigating the influence of the test media on the LoD_95_^IS*6110*^ of the test assays, saline yielded significantly higher values with all assays than human sputum and MUCAS (p≤0.01), while MUCAS yielded only with BD-MAX slightly higher LoD_95_^IS*6110*^ values in MUCAS than in human sputum (p≤0.05).

### Sensitivity of Rif and Inh resistance marker detection is decreased

The analytical sensitivities of BD-MAX and XP-Ultra with regards to the detection of genetic resistance markers (reported as LoD_95_^HR^; H = Inh, R = Rif) were lower than the detection of MTB (Table 1, S2 and S3 Figs). With H37Rv, XP-Ultra and BD-MAX validly indicated Rif (and Inh) susceptibility at 1.8 to 9.2 times and 4.9 to 48.4 times, respectively, higher bacterial concentrations than needed to validly detect MTBC. Both, LoD_95_^Rif^ and LoD_95_^Inh^ were lower in saline and higher in MUCAS than in human sputum.

**Table 1:** Analytical sensitivity for the detecting genetic resistance markers of Inh (H) and Rif (R) reported as LoD95HR of BD-MAX and XP-Ultra determined by mathematical interpolation.

	BD-MAX cfu/ml (CI_95%_)					XP-Ultra cfu/ml (CI_95%_)		
Matrix	MTB	Rif		Inh		MTB	Rif	
	*H37Rv (WT)*	*H37Rv (WT)*	*T837 (MDR)*	*H37Rv (WT)*	*T837 (MDR)*	*H37Rv (WT)*	*H37Rv (WT)*	*T837 (MDR)*
**Sputum**	2.1 (0.9–23.3)	7.8 (2.7–462.8)	17.6 (9.0–477.6)	19.4 (5.8–993.4)	17.6 (9.0–477.6)	3.1 (1.2–88.9)	49.3 (11–9,544)	63.2 (33.5–271.4)
**MUCAS**	6.3 (2.9–31.8)	44.1 (12.3–2,104)	1,388.2 (53.3-NA)	2,652.8 (1381–10,171)	212.8 (30.0-NA)	1.5 (0.7–5.0)	72.6 (26–609)	194.8 (54.9–27,421)
**Saline**	2.3 (1.1–12.0)	5.3 (2.1–124.6)	7.7 (4.6–16.5)	4.1 (1.6–228.3)	7.7 (4.6–16.5)	11.5 (5.6–47.3)	56.5 (24.3–272)	2,165.9 (591–35,996)

In order to verify that the LoD_95_^HR^ were reproducible for resistant strains, we twice tested MDR-TB strain T837 which harbors the most frequent resistance mutations *rpoB* S531L and *katG* S315T. In human sputum, LoD_95_^HR^ values of BD-MAX and XP-Ultra were within the same ranges with both strains, H37Rv and T837 (Table 1, S2 and S3 Figs). T837 yielded high LoD_95_^HR^ values for Rif with XP-Ultra in all matrices and with BD-MAX in saline and MUCAS, respectively; H37Rv yielded high LoD_95_^HR^ values for Inh with BD-MAX in MUCAS, however, 95% confidence intervals were wide.

## Discussion

Recently launched PCR assays including BD-MAX, XP-Ultra and FluoroType MTBDR allow microbiologists to choose from a larger variety of improved TB diagnostics. Besides framework conditions like spectrum of analysis, investment, running costs and practicability, the sensitivity is one of the major criteria driving the decision for a specific test. However, sensitivity rates reported by manufacturers and authors of evaluation studies are rarely comparable because test conditions, particularly test matrices and bacterial concentrations of test samples, can strongly deviate from one another. With our study we provide not only a direct head-to-head comparison of analytical sensitivities of two prominent representatives of the new generation of TB assays, we also propose a standardized evaluation plan of analytic sensitivity using an artificial sputum with precisely defined bacterial concentrations.

When using FT-MTB as a well-established representative of the recent TB-PCR generation, both BD-MAX and XP-Ultra showed significantly higher analytical sensitivity for MTBC. Only FT-MTB requires NALC-NaOH decontamination before DNA release; therefore, the loss of bacteria and / or free DNA before amplification might account for the lower sensitivity as it has been observed that NALC-NaOH decontamination reduces colony counts [23]. When we correlated the numbers of IS*6110* copies with the colony counts in this study, the presence of free DNA or dead bacteria became obvious: one cfu of H37Rv corresponded to more than eleven thousand IS*6110* copies in bacterial suspensions before spiking the test matrices. As H37Rv contains sixteen IS*6110* elements [24], we observed on average 736 MTB genomes per cfu. Since we have filtered the bacterial suspensions through 5 μm pores before plating for cfu counts, we hypothesize that 1 cfu originated only from one to very few viable bacterial cells. Free DNA might have partially resulted from osmotic cell rupture in saline solutions or from NaOH induced denaturation [25]. During decontamination, free DNA would be washed off with the effect that total DNA content introduced in the FT-MTB assay would be significantly lower than in XP-Ultra and BD-MAX resulting in potentially biased LoD_95_ values of FT-MTB in such type of *in vitro* studies. Whether decreased sensitivity due to DNA loss during sample decontamination has a clinical impact needs to be further investigated. One should be also aware that free DNA in spiked samples might artificially lower the limit of detection. In a previous study, the LoD_95_^TB^ value of XP-Ultra was reported to be 15.6 cfu/ml [14], higher than our measured LoD_95_^TB^ of 1.5 to 11.5 cfu/ml (depending on the matrix). However, free DNA has equally lowered LoD_95_ values of both XP-Ultra and BD-MAX in our system; therefore, a direct comparison of analytical sensitivities can be made. To overcome the bias of free DNA, future investigations of the diagnostic sensitivity should include sputum samples of relevant TB-patient groups.

Although BD-MAX and XP-Ultra yield comparable analytical sensitives, slight inter-test variations were observed using different test matrices. For BD-MAX, MTB detection increased 3-fold in MUCAS compared to human sputum and declined two-fold by XP-Ultra. In addition, the LoD_95_ for MTB detection in saline was almost identical as in human sputum by BD-MAX, but increased nearly 4-fold by XP-Ultra. The difference in lysis chemistries and DNA extraction procedures applied by both assays might account for this observation.

Rapid detection of antibiotic resistances is essential for early and appropriate treatment initiation. With the upgrade from XP MTB/RIF to XP-Ultra, Cepheid has markedly increased the assay’s sensitivity which is mainly noticeable at very low bacterial concentrations identified by the assay as ‘trace call’ [26]. Additionally, at such low concentrations XP-Ultra flags Rif resistance markers as ‘indeterminate’. In our recent evaluation of the ABBOTT RealTime MTB INH/RIF, we observed a high rate of indeterminate resistance markers for both Rif and Inh, when smear microscopy was scanty or negative [17]. Zimmermann et al observed 8.9% of indeterminate Rif/Inh results with BD-MAX in 518 clinical samples [15] which is in line with the before mentioned publications on Xpert MTB/RIF [14] and Abbott RealTime MTB RIF/INH [17]. This confirms that molecular resistance profiling is less sensitive compared to MTBC detection and coincides with our findings that XP-Ultra’s and BD-MAX’s LoD_95_^HR^ were higher than its LoD_95_^TB^, especially when saline or MUCAS were used albeit not being approved by the manufacturer. MUCAS was inferior to human sputum when antibiotic resistance was tested using BD-MAX, which might indicate a more limited use for monitoring resistance. However, only a slightly decreased analytical sensitivity for Rif resistance screening was observed in MUCAS by XP-Ultra. This is in line with a recent study using TB/MDR-TB sputum samples showing that indeterminate results are obtained more frequently with BD-MAX than XP-Ultra for the detection of drug resistance [27]. Nevertheless, BD-MAX detected MTBC as well as antibiotic resistance with high sensitivity and specificity comparable to Xpert MTB/RIF in a clinical multicenter study in South Africa, Uganda, India and Peru [28].

With this study, we have also introduced a novel fully standardized mucin based artificial sputum for the evaluation and inter-assay comparison of the analytical sensitivity of diagnostic assays. We demonstrated that NAAT-based diagnostic assays performed mostly comparable with spiked MUCAS and pooled human sputum, but differently with saline. Since human sputum is not always obtainable and highly heterogeneous [29–32], test results and inter-laboratory and inter-study comparability of results are impossible. MUCAS is chemically very similar to and behaves like human sputum in molecular assays. It is easily manufacturable and always reproducible in required quantities and can serve as an alternative test matrix for any kind of evaluation, validation or verification of molecular TB assays applied to respiratory specimens. By adhering to the manufacturers’ instructions, using identical test matrices which are produced and spiked following the same protocol, and calculating LoD_95_ values using the same algorithms, analytical sensitivity values will become fully comparable even when determined at different times and places. This would be tremendously helpful for laboratory experts to make the right decision for or against an assay which might otherwise fulfil all framework conditions.

It was beyond the scope of this study to include more clinical isolates with relevant mutations causing Inh and Rif resistance [33]; however this should be considered in future research. Diagnostic sensitivities using sputum from patients were also not investigated. This paper provides a pre-clinical evaluation plan for analytical sensitivities of molecular diagnostics.

## Supporting information

S1 Fig Correlation of IS*6110* insertion elements with cfu’s. (TIF)

S2 Fig LoD_95_^HR^ (detection of Inh and/or Rif resistance markers) of BD-MAX, XP-Ultra, and FT-MTB in human sputum (A), MUCAS (B), and physiological saline solution (C) using MTB reference strain H37Rv.
(TIF)

S3 Fig LoD_95_^HR^ (detection of Inh and/or Rif resistance markers) of BD-MAX, XP-Ultra, and FT-MTB in human sputum (A), MUCAS (B), and physiological saline solution (C) using clinical MDR strain T837.
(TIF)
