# Supplementary figures and images for: A pre-clinical validation plan to evaluate analytical sensitivities of molecular diagnostics such as BD MAX MDR-TB, Xpert MTB/Rif Ultra and FluoroType MTB
Source: PLoS One. 2020 Jan 7;15(1):e0227215. doi: 10.1371/journal.pone.0227215 (PMC6946130; doi:10.1371/journal.pone.0227215)

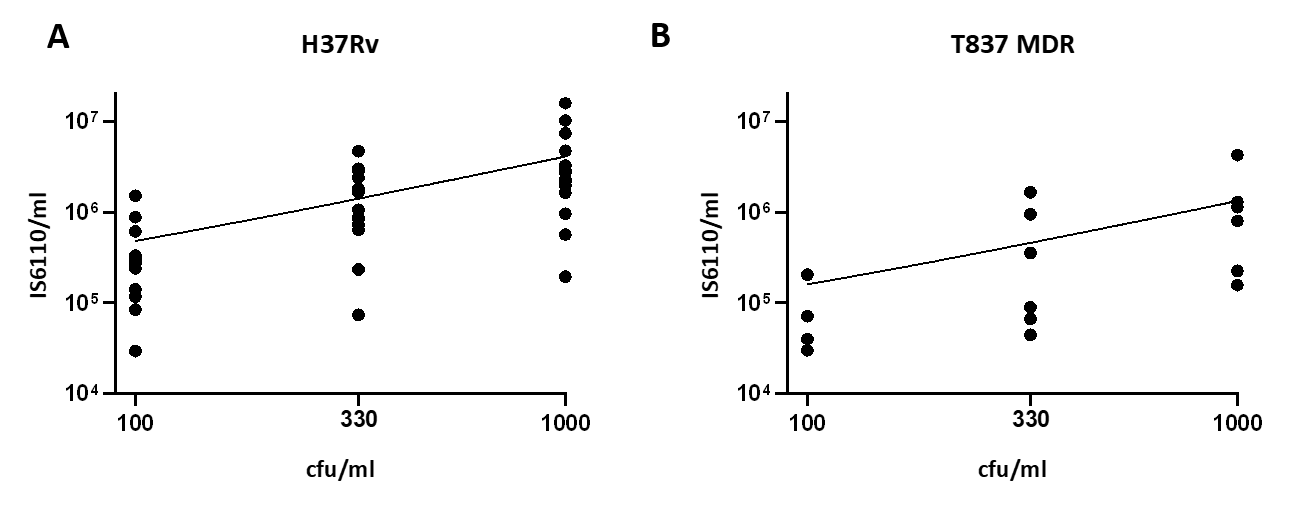

Supplement: S1 Fig — (TIF) [file pone.0227215.s001.tif]

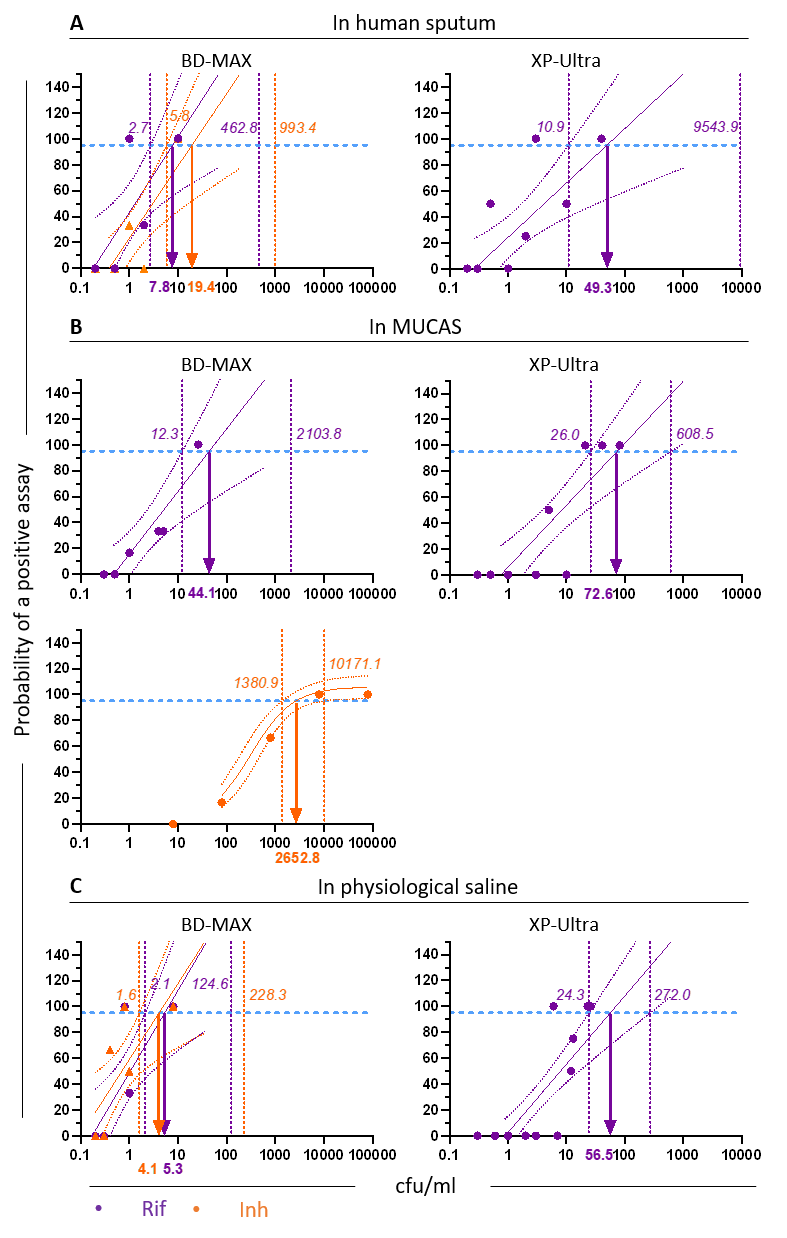

Supplement: S2 Fig — LoD95HR (detection of Inh and/or Rif resistance markers) of BD-MAX, XP-Ultra, and FT-MTB in human sputum (A), MUCAS (B), and physiological saline solution (C) using MTB reference strain H37Rv. (TIF) [file pone.0227215.s002.tif]

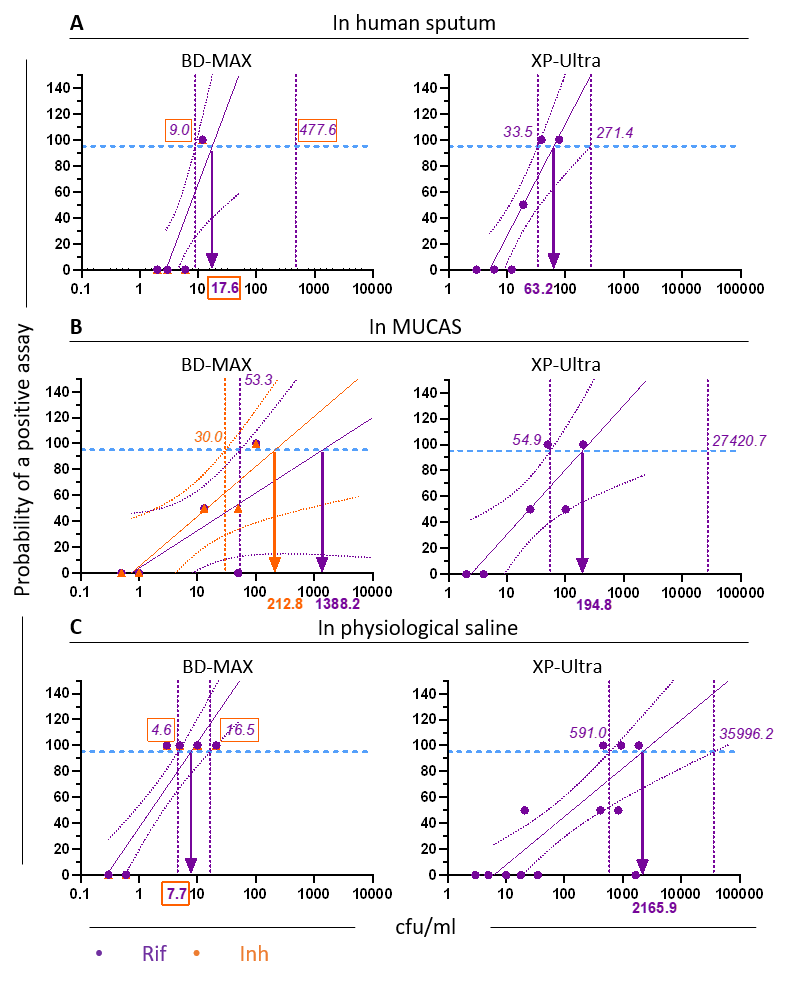

Supplement: S3 Fig — LoD95HR (detection of Inh and/or Rif resistance markers) of BD-MAX, XP-Ultra, and FT-MTB in human sputum (A), MUCAS (B), and physiological saline solution (C) using clinical MDR strain T837. (TIF) [file pone.0227215.s003.tif]
